# Solution of the Nonlinear Mixed Volterra-Fredholm Integral Equations by Hybrid of Block-Pulse Functions and Bernoulli Polynomials

**DOI:** 10.1155/2014/413623

**Published:** 2014-01-12

**Authors:** S. Mashayekhi, M. Razzaghi, O. Tripak

**Affiliations:** ^1^Department of Mathematics and Statistics, Mississippi State University, MS 39762, USA; ^2^Department of Mathematics and Statistics, Faculty of Science, Prince of Songkla University, Songkhla 90112, Thailand

## Abstract

A new numerical method for solving the nonlinear mixed Volterra-Fredholm integral equations is presented. This method is based upon hybrid functions approximation. The properties of hybrid functions consisting of block-pulse functions and Bernoulli polynomials are presented. The operational matrices of integration and product are given. These matrices are then utilized to reduce the nonlinear mixed Volterra-Fredholm integral equations to the solution of algebraic equations. Illustrative examples are included to demonstrate the validity and applicability of the technique.

## 1. Introduction

There is considerable literature that discusses approximating the solution of linear and nonlinear Hammerstein integral equations [1–6]. For Fredholm-Hammerstein integral equations, the classical method of successive approximations was introduced in [1]. A variation of the Nystrom method was presented in [2], and a collocation-type method was developed in [3]. In [4], Brunner applied a collocation-type method to nonlinear Volterra-Hammerstein integral equations and integrodifferential equations and discussed its connection with the iterated collocation method. Han [5] introduced and discussed the asymptotic error expansion of a collocation-type method for Volterra-Hammerstein integral equations. The existence of solutions to nonlinear Hammerstein integral equations was discussed in [6]. Further, Reihani and Abadi [7] and Hsiao [8] applied rationalized Haar functions and hybrid of block-pulse functions and Legendre polynomials, respectively, for solving Fredholm and Volterra integral equations of the second kind. Several authors consider the nonlinear mixed Volterra-Fredholm integral equations of the form

(1) y(t)=f(t)+λ1∫0tκ1(t,s)g1(s,y(s))ds +λ2∫01κ2(t,s)g2(s,y(s))ds, 0≤t, s≤1,

where *λ*_1_ and *λ*_2_ are constants and *f*(*t*) and the kernels *κ*_1_(*t*, *s*) and *κ*_2_(*t*, *s*) are given functions assumed to have *n*th derivatives on the interval 0 ≤ *x*, *t* ≤ 1. For the case *g*_1_(*s*, *y*(*s*)) = *y* ^*p*^(*s*) and *g*_2_(*s*, *y*(*s*)) = *y* ^*q*^(*s*), where *p* and *q* are nonnegative integers, Yalçinbaş [9], Bildik and Inc [10], and Hashemizadeh et al. [11] used Taylor series, modified decomposition method, and hybrid of block-pulse functions and Legendre polynomials, respectively, to find the solution. For the case *g*_1_(*s*, *y*(*s*)) = *F*_1_(*y*(*s*)) and *g*_2_(*s*, *y*(*s*)) = *F*_2_(*y*(*s*)), where *F*_1_(*y*(*s*)) and *F*_2_(*y*(*s*)) are given continuous functions which are nonlinear with respect to *y*(*s*), Yousefi and Razzaghi [12] applied Legendre wavelets to obtain the solution, and for the general case, where *g*_1_(*s*, *y*(*s*)) and *g*_1_(*s*, *y*(*s*)) are given continuous functions which are nonlinear with respect to *s* and *y*(*s*), Ordokhani [13] and Marzban et al. [14] applied the rationalized Haar functions and hybrid of block-pulse functions and Lagrange polynomials, respectively, to get the solution.

The available sets of orthogonal functions can be divided into three classes. The first class includes sets of piecewise constant basis functions (PCBF's) (e.g., block-pulse, Haar, and Walsh). The second class consists of sets of orthogonal polynomials (e.g., Chebyshev, Laguerre, and Legendre). The third class is the set of sine-cosine functions in the Fourier series.

Orthogonal functions have been used when dealing with various problems of the dynamical systems. The approach is based on converting the underlying differential equation into an integral equation through integration, approximating various signals involved in the equation by truncated orthogonal functions, and using the operational matrix of integration *P* to eliminate the integral operations. The matrix *P* can be uniquely determined based on the particular orthogonal functions (see [15] and references therein). Among orthogonal polynomials, the shifted Legendre polynomial is computationally more effective [16]. The Bernoulli polynomials and Taylor series are not based on orthogonal functions; nevertheless, they possess the operational matrices of integration. However, since the integration of the cross product of two Taylor series vectors is given in terms of a Hilbert matrix [17], which is known to be ill-conditioned, the applications of Taylor series are limited.

Recently, different types of hybrid functions have been used for solving integral equations and proved to be a mathematical power tool [8, 11, 14].

In the present paper we introduce a new direct computational method to solve nonlinear mixed Volterra-Fredholm integral equations in (1). We approximate the solution not to the equation in its original form but rather to an equivalent equation *z*_1_(*t*) = *g*_1_(*t*, *y*(*t*)) and *z*_2_(*t*) = *g*_2_(*t*, *y*(*t*)), *t* ∈ [0,1]. The functions *z*_1_(*t*) and *z*_2_(*t*) are approximated by hybrid functions with unknown coefficients. These hybrid functions, which consist of block-pulse functions and Bernoulli polynomials together with their operational matrices of integration and product, are given. These matrices are then used to evaluate the coefficients of the hybrid functions for solution of nonlinear mixed Volterra-Fredholm integral equations.

The outline of this paper is as follows. In Section 2, we introduce properties of Bernoulli polynomials and hybrid functions. In Section 3, the numerical method is used to approximate the nonlinear mixed Volterra-Fredholm integral equations, and in Section 4, we report our numerical findings and demonstrate the accuracy of the proposed numerical scheme by considering five numerical examples.

## 2. Hybrid Functions

### 2.1. Properties of Bernoulli Polynomials

The Bernoulli polynomials of order *m* are defined in [18] by

(2) $$\begin{array}{c} \beta_{m}\left(t\right)=\overset{m}{\underset{k=0}{\sum }}\left(\begin{array}{c} m\\ k \end{array}\right)\alpha_{k}t^{m-k}, \end{array}$$

where *α*_*k*_, *k* = 0,1,…, *m* are Bernoulli numbers. These numbers are a sequence of signed rational numbers, which arise in the series expansion of trigonometric functions [19] and can be defined by the identity

(3) $$\begin{array}{c} \frac{t}{e^{t}-1}=\overset{\infty }{\underset{n=0}{\sum }}\alpha_{n}\frac{t^{n}}{n!}. \end{array}$$

The first few Bernoulli numbers are

(4) $$\begin{array}{c} \alpha_{0}=1,\;\;\alpha_{1}=-\frac{1}{2},\;\;\alpha_{2}=\frac{1}{6},\;\;\alpha_{4}=-\frac{1}{30}, \end{array}$$

with *α*_2*k*+1_ = 0, *k* = 1,2, 3,….

The first few Bernoulli polynomials are

(5) $$\begin{array}{c} \beta_{0}\left(t\right)=1,\;\;\beta_{1}\left(t\right)=t-\frac{1}{2},\\ \beta_{2}\left(t\right)=t^{2}-t+\frac{1}{6},\;\;\beta_{3}\left(t\right)=t^{3}-\frac{3}{2}t^{2}+\frac{1}{2}t. \end{array}$$

According to [20], Bernoulli polynomials form a complete basis over the interval [0,1].

## 3. Hybrid of Block-Pulse Functions and Bernoulli Polynomials

Hybrid functions *b*_*nm*_(*t*), *n* = 1,2,…, *N*, *m* = 0,1,…, *M* are defined on the interval [0, *t*_*f*_] as [21]

(6) $$\begin{array}{c} b_{nm}\left(t\right)=\{\begin{array}{cc} \beta_{m}\left(\frac{N}{t_{f}}t-n+1\right), & t\in \left[\frac{n-1}{N}t_{f},\frac{n}{N}t_{f}\right],\\ 0, & \text{otherwise}, \end{array} \end{array}$$

where *n* and *m* are the order of block-pulse functions and Bernoulli polynomials, respectively.

### 3.1. Function Approximation

Let *H* = *L* ^2^[0,1], and assume that {*b*_10_(*t*), *b*_20_(*t*),…, *b*_*NM*_(*t*)} ⊂ *H* is the set of hybrid of block-pulse functions and Bernoulli polynomials, and

(7) Y=span{b10(t),b20(t),…,bN0(t),b11(t),b21(t),…,bN1(t),…,b1M(t),b2M(t),…,bNM(t)},

with *f* being an arbitrary element in *H*. Since *Y* is a finite dimensional vector space, *f* has the unique best approximation out of *Y* such as *f*_0_ ∈ *Y*, that is,

(8) $$\begin{array}{c} \forall y\in Y,\;\;||f-f_{0}||\leq ||f-y||. \end{array}$$

Since *f*_0_ ∈ *Y*, there exist unique coefficients *c*_10_, *c*_20_,…, *c*_*NM*_ such that

(9) $$\begin{array}{c} f≃f_{0}=\overset{M}{\underset{m=0}{\sum }}\;‍\overset{N}{\underset{n=1}{\sum }}c_{nm}b_{nm}\left(t\right)=C^{T}B\left(t\right), \end{array}$$

where

(10) BT(t)=[b10(t),b20(t),…,bN0(t),b11(t),b21(t),…,bN1(t),…,b1M(t),b2M(t),…,bNM(t)],

(11) CT=[c10,c20,…,cN0,c11,c21,…,cN1,…,c1M,c2M,…,cNM].

Further, by using (9), we can obtain

(12) $$\begin{array}{c} D=\overset{1}{\underset{0}{\int }}B\left(t\right)B^{T}\left(t\right)dt, \end{array}$$

where *D* is a sparse invertible matrix [21].

### 3.2. Function of Two Variables Approximation

Let *g*(*t*, *s*) be a function of two independent variables defined for *t* ∈ [0,1] and *s* ∈ [0,1]. Then *g* can be expanded as

(13) $$\begin{array}{c} g\left(t,s\right)=B^{T}\left(t\right)\bar{G}B\left(s\right)=\overset{M}{\underset{j=0}{\sum }}\;‍\overset{N}{\underset{i=1}{\sum }}\kappa_{ij}\left(t\right)b_{ij}\left(s\right). \end{array}$$

Let the matrix $\bar{G}$ be given by

(14) $$\begin{array}{c} \bar{G}=\left[\begin{array}{cccc} \varphi_{10}^{10} & \varphi_{20}^{10} & \ldots & \varphi_{NM}^{10}\\ \varphi_{10}^{20} & \varphi_{20}^{20} & \ldots & \varphi_{NM}^{20}\\ \vdots & \vdots & \vdots & \vdots \\ \varphi_{10}^{NM} & \varphi_{20}^{NM} & \ldots & \varphi_{NM}^{NM} \end{array}\right]. \end{array}$$

From (13), we get

(15) [κ10(t),κ20(t),…,κNM(t)]D  =[∫01g(t,s)b10(s)ds,    ∫01g(t,s)b20(s)ds,…,∫01g(t,s)bNM(s)ds].

Also, *κ*_*ij*_(*t*) can be expanded as

(16) $$\begin{array}{c} \kappa_{ij}\left(t\right)=\overset{M}{\underset{q=0}{\sum }}\;‍\overset{N}{\underset{p=1}{\sum }}\varphi_{pq}^{ij}b_{pq}\left(t\right), \end{array}$$

where *φ*_*pq*_^*ij*^ can be obtained similar to (15).

### 3.3. Operational Matrices of Integration and Product

The integration of the hybrid functions *B*(*t*) defined in (10) is given by

(17) $$\begin{array}{c} \overset{t}{\underset{0}{\int }}B\left(t^{\prime }\right)dt^{\prime }≃PB\left(t\right), \end{array}$$

where *P* is the *N*(*M* + 1) × *N*(*M* + 1) operational matrix of integration. The product of two hybrid functions with the vector *C* is given by

(18) $$\begin{array}{c} B\left(t\right)B^{T}\left(t\right)C≃\tilde{C}B\left(t\right), \end{array}$$

where $\tilde{C}$ is the *N*(*M* + 1) × *N*(*M* + 1) product operational matrix. The matrices *P* and $\tilde{C}$ are given in [22].

### 3.4. Approximation Errors

In this section we obtain bounds for the error of best approximation in terms of Sobolev norms. This norm is defined in the interval (*a*, *b*) for *μ* ≥ 0 by

(19) ||f||Hμ(a,b)=(∑k=0μ∫ab|f(k)(x)|2dx)1/2=(∑k=0μ||f(k)||L2(a,b)2)1/2,

where *f* ^(*k*)^ denotes the *k*th derivative of *f*. The symbol |*f*|_*H*^*μ*;*M*^(0,1)_ which is introduced in [23] is defined by

(20) $$\begin{array}{c} {\left|f\right|}_{H^{\mu ;M}(0,1)}={\left(\overset{\mu }{\underset{k=\min(\mu ,M+1)}{\sum }}{||f^{\left(k\right)}||}_{L^{2}\left(0,1\right)}^{2}\right)}^{1/2}. \end{array}$$

**Theorem 1**

Suppose that *f* ∈ *H* ^*μ*^(0,1) with *μ* ≥ 0. If *P*_*M*_ *f* = ∑_*m*=0_^*M*^ *c*_*m*_ *β*_*m*_ is the best approximation of *f* then

(21) $$\begin{array}{c} {||f-P_{M}f||}_{L^{2}(0,1)}\leq cM^{-\mu }{\left|f\right|}_{H^{\mu ;M}(0,1)} \end{array}$$

and, for 1 ≤ *r* ≤ *μ*,

(22) $$\begin{array}{c} {||f-P_{M}f||}_{H^{r}(0,1)}\leq cM^{2r-(1/2)-\mu }{\left|f\right|}_{H^{\mu ;M}(0,1)}, \end{array}$$

where *c* depends on *μ*.

**Proof**

Let *f* ∈ *H* ^*μ*^(0,1) with *μ* ≥ 0 and let ∑_*m*=0_^*M*^ *c*_*m*_′*p*_*m*_ be the best approximation of *f*, which is constructed by using shifted Legendre polynomials *p*_*m*_, *m* = 0,…, *M* in the interval [0,1]. Then [23]

(23) $$\begin{array}{c} {||f-\overset{M}{\underset{m=0}{\sum }}c_{m}^{\prime }p_{m}||}_{L^{2}(0,1)}\leq cM^{-\mu }{\left|f\right|}_{H^{\mu ;M}(0,1)}, \end{array}$$

and, for 1 ≤ *r* ≤ *μ*,

(24) $$\begin{array}{c} {||f-\overset{M}{\underset{m=0}{\sum }}c_{m}^{\prime }p_{m}||}_{H^{r}(0,1)}\leq cM^{2r-(1/2)-\mu }{\left|f\right|}_{H^{\mu ;M}(0,1)}. \end{array}$$

Since the best approximation is unique [20], we have

(25) ||f−∑m=0Mcm′pm||L2(0,1)=||f−PMf||L2(0,1),||f−∑m=0Mcm′pm||Hr(0,1)=||f−PMf||Hr(0,1),

and by using (23)–(25) we can obtain (21) and (22).

## 4. The Numerical Method

We approximate (1) as follows.

Define

(26) $$\begin{array}{c} z_{1}\left(t\right)=g_{1}\left(t,y\left(t\right)\right),\;z_{2}\left(t\right)=g_{2}\left(t,y\left(t\right)\right),\;t\in \left[0,1\right]. \end{array}$$

By using (1) and (26), we have

(27) z1(t)=g1(t,f(t)+λ1∫0tκ1(t,s)z1(s)ds +λ2∫01κ2(t,s)z2(s)ds),z2(t)=g2(t,f(t)+λ1∫0tκ1(t,s)z1(s)ds +λ2∫01κ2(t,s)z2(s)ds).

By using (9) and (13) we get

(28) $$\begin{array}{c} z_{1}\left(t\right)=C_{1}^{T}B\left(t\right),\;\;z_{2}\left(t\right)=C_{2}^{T}B\left(t\right),\\ \kappa_{1}\left(t,s\right)=B^{T}\left(t\right)K_{1}B\left(s\right),\;\;\kappa_{2}\left(t,s\right)=B^{T}\left(t\right)K_{2}B\left(s\right). \end{array}$$

By substituting (28) in (27) we have

(29) C1TB(t)=g1(t,f(t)+λ1∫0tBT(t)K1B(s)BT(s)C1ds +λ2∫01BT(t)K2B(s)BT(s)C2ds),C2TB(t)=g2(t,f(t)+λ1∫0tBT(t)K1B(s)BT(s)C1ds +λ2∫01BT(t)K2B(s)BT(s)C2ds).

By using (12), (17), and (18) we get

(30) C1TB(t)=g1(t,f(t)+λ1BT(t)K1C~1PB(t) +λ2BT(t)K2DC2),C2TB(t)=g2(t,f(t)+λ1BT(t)K1C~1PB(t)+ λ2BT(t)K2DC2).

We collocate (30) at Newton-cotes nodes *t*_*i*_

(31) $$\begin{array}{c} t_{i}=\frac{i+1}{2N\left(M+1\right)},\;i=0,1,\ldots ,2N\left(M+1\right)-2. \end{array}$$

So we have

(32) C1TB(ti)=g1(ti,f(ti)+λ1BT(ti)K1C~1PB(ti)+λ2BT(ti)K2DC2),C2TB(ti)=g2(ti,f(ti)+λ1BT(ti)K1C~1PB(ti)+λ2BT(ti)K2DC2),

for *i* = 0,1,…, 2*N*(*M* + 1) − 2.

Equation (32) can be solved for the unknown *C*_1_ and *C*_2_. The required approximations to the solution *y*(*t*) in (1) are given by

(33) y(t)=f(t)+λ1∫0tκ1(t,s)z1(s)ds +λ2∫01κ2(t,s)z2(s)ds, 0≤t≤1.

## 5. Illustrative Example

In this section, five examples are given to demonstrate the applicability and accuracy of our method.  Example 1 is a nonlinear mixed Volterra-Fredholm-Hammerstein integral equation which was considered in [13] by using rationalized Haar functions (RHF) and also solved in [24] by applying Chebyshev approximation. Examples 2 and 3 are the integral equations reformulation of the nonlinear two-point boundary value problems considered in [13, 25] by using RHF and Adomian method, respectively. Although the reformulated integral equations in Examples 2 and 3 are Fredholm-Hammerstein integral equations, the method described here can be used. Examples 1–3 were also solved in [14] by using hybrid of block-pulse functions and Lagrange polynomials. For Examples 1–3, we compare our findings with the numerical results in [14] which have been shown to be superior to those of [13, 24, 25]. Examples 4 and 5 are Fredholm and Volterra integral equations of the second kind, respectively, which were considered in [7] by using RHF which were also solved in [8] by using hybrid of block-pulse functions and Legendre polynomials. For Examples 4 and 5, we compare our findings with the numerical results in [8] which have been shown to be superior to those of [7].

For approximating an arbitrary time function, the advantages of Bernoulli polynomials *β*_*m*_(*t*), *m* = 0,1, 2,…, *M* where 0 ≤ *t* ≤ 1, over shifted Legendre polynomials *p*_*m*_(*t*), *m* = 0,1, 2,…, *M*, where 0 ≤ *t* ≤ 1, are given in [21] and over Lagrange polynomials *Ł*_*m*_(*t*), *m* = 0,1, 2,…, *M*, where 0 ≤ *t* ≤ 1, are given below.

*Advantages of Bernoulli Polynomials over Lagrange Polynomials.* (a) The operational matrix of integration *P* in Bernoulli polynomials has less error than *P* for Lagrange polynomials. This is because, for *P* in *β*_0_(*t*), *β*_1_(*t*),…, *β*_*M*_(*t*), we ignore the term from the integration of *β*_*M*_(*t*), while for *P* in Lagrange polynomials in *L*_0_(*t*), *L*_1_(*t*),…, *L*_*M*_(*t*) we ignore the terms from the integration of each of *L*_*m*_(*t*), *m* = 0,1,…, *M*.

(b) Bernoulli polynomials have less terms than Lagrange polynomials. For example, *β*_0_(*t*), *β*_1_(*t*), *β*_2_(*t*), *β*_3_(*t*), *β*_4_(*t*), *β*_5_(*t*),  and *β*_6_(*t*) have 1,2, 3,3, 4,4, and 5 terms, respectively, while *L*_0_(*t*), *L*_1_(*t*),…, *L*_6_(*t*) all have 7 terms, and this difference will increase by increasing *m*. Hence, for approximating an arbitrary function, we use less CPU time by applying Bernoulli polynomials as compared to Lagrange polynomials.

(c) The coefficients of individual terms in Bernoulli polynomials *β*_*m*_(*t*) are smaller than the coefficient of individual terms in the Lagrange polynomials *L*_*m*_(*t*). Since the computational errors in the product are related to the coefficients of individual terms, the computational errors are less by using Bernoulli polynomials.

**Example 1**

Consider the integral equation given in [13] by

(34) y(t)=2cost−2+3∫0tsin(t−s)cos2sds +67−6cos1∫01(1−s)cos2t(s+y(s))ds, 0≤t<1.

The exact solution is *y*(*t*) = cos*t*.

For this integral equation we choose *N* = 1 and *M* = 4.

Let

(35) z(t)=y(t)=WTB(t),

(36) sin(t−s)cos2s=BT(t)Q1B(s),(1−s)cos2ts=BT(t)Q2B(s),

(37) (1−s)cos2t=BT(t)Q3B(s),

where *Q*_1_, *Q*_2_, and *Q*_3_ are obtained similar to (13). By substituting (35)–(37) in (34) we have

(38) WTB(t)=2cos(t)−2+3BT(t)Q1PB(t) +67−6cos1(BT(t)Q2PB(1)+BT(t)Q3D1W),

where *D*_1_ can be calculated similar to (12). We collocate (38) at

(39) $$\begin{array}{c} t_{i}=\frac{i+1}{10},\;i=0,1,\ldots ,8. \end{array}$$

We get

(40) WTB(ti)=2cos(ti)−2+3BT(ti)Q1PB(ti)+67−6cos1 ×(BT(ti)Q2PB(1)+BT(ti)Q3D1W),i=0,1,…,8.

Solving (40) we obtain *W* ^*T*^ in (35).  Table 1 shows the absolute errors of exact and approximate solutions in some points of the interval [0,1] obtained by the present method for *N* = 1 and *M* = 4 together with the method of [14]. In this Table *M*_1_ is order of Lagrange polynomials.

**Example 2**

Consider

(41) $$\begin{array}{c} y^{\prime \prime }\left(t\right)-e^{y(t)}=0,\;0\leq t\leq 1,\\ y\left(0\right)=y\left(1\right)=0, \end{array}$$

which is of great interest in hydrodynamics [26]. This problem has the unique solution [3]

(42) $$\begin{array}{c} y_{e}\left(t\right)=-\ln\left(2\right)+\ln\left(\lambda \left(t\right)\right), \end{array}$$

where

(43) $$\begin{array}{c} \lambda \left(t\right)=\left(\frac{c}{\cos\left(\left(1/2\right)c\right)\left(t-\left(1/2\right)\right)}\right). \end{array}$$

Here, *c* is the root of the equation

(44) $$\begin{array}{c} {\left(\frac{c}{\cos\left(c/4\right)}\right)}^{2}=2. \end{array}$$

Equation (41) can be reformulated as the integral equation

(45) $$\begin{array}{c} y\left(t\right)=\overset{1}{\underset{0}{\int }}k\left(t,s\right)e^{y(s)}ds,\;0\leq t\leq 1, \end{array}$$

where

(46) $$\begin{array}{c} k\left(t,s\right)=\{\begin{array}{cc} -s\left(1-t\right), & s\leq t,\\ -t\left(1-s\right), & t\leq s. \end{array} \end{array}$$

Table 2 represents the computational results of the errors ||*y*−*y*_*e*_||_*L*^*∞*^(0,1)_ for different values of *N* and *M* obtained by the present method together with different values of *N* and *M*_1_ in [14]. In Table 2, *y* and *y*_*e*_ denote the approximate and exact solutions, respectively.

**Example 3**

In this example we consider the mathematical model for an adiabatic tubular chemical reactor discussed in [27, 28], which, in the case of steady state solutions, can be stated as the ordinary differential equation

(47) $$\begin{array}{c} y^{\prime \prime }\left(t\right)-\lambda y^{\prime }\left(t\right)+\lambda \mu \left(\beta -y\left(t\right)\right)e^{y(t)}=0,\;0\leq t\leq 1,\\ y^{\prime }\left(0\right)=\lambda y\left(0\right),\;y^{\prime }\left(1\right)=0. \end{array}$$

The problem can be converted into a Hammerstein integral equation of the form [28]

(48) $$\begin{array}{c} y\left(t\right)=\overset{1}{\underset{0}{\int }}k\left(t,s\right)G\left(s,y\left(s\right)\right)ds,\;0\leq t\leq 1, \end{array}$$

where *k*(*x*, *t*) is defined by

(49) $$\begin{array}{c} k\left(t,s\right)=\{\begin{array}{cc} 1, & s\leq t,\\ e^{\lambda (t-s)}, & t\leq s, \end{array}\\ G\left(s,y\left(s\right)\right)=\mu \left(\beta -y\left(s\right)\right)e^{y(s)}. \end{array}$$

The existence and uniqueness of the solution for this Hammerstein integral equation with respect to the value of parameters *λ*,  *μ*, and *β* are given in [28]. In [25] the Adomian method is used to solve the integral equation (48) for the particular values of the parameters *λ* = 10,  *μ* = 0.02, and *β* = 3 which guarantee the existence and uniqueness of the solution for this integral equation [28].

Table 3 gives a comparison between the numerical results of *y*(*t*) in some points of the interval [0,1] obtained by the Adomian method given in [25], together with the method in [14] for *N* = 4 and *M*_1_ = 7 and by the present method for *N* = 4 and *M* = 4.

**Example 4**

Consider the Fredholm integral equation of the second kind [7]

(50) $$\begin{array}{c} y\left(t\right)=e^{2t+(1/3)}+\overset{1}{\underset{0}{\int }}-\frac{1}{3}e^{2t-(5/3)s}y\left(s\right)ds, \end{array}$$

with the exact solution *y*(*t*) = *e* ^2*t*^.  Table 4 gives a comparison between the numerical results of *y*(*t*) in some points of the interval [0,1] obtained by the method given in [8] for *N* = 2 and *M*_3_ = 11 and by the present method for *N* = 2 and *M* = 4. In this Table *M*_3_ is order of Legendre polynomials.

**Example 5**

Consider the Volterra linear integral equation of the second kind [7]

(51) $$\begin{array}{c} y\left(t\right)=\cos\left(t\right)-\overset{t}{\underset{0}{\int }}\left(t-s\right)\cos\left(t-s\right)y\left(s\right)ds, \end{array}$$

with the exact solution $y(t)=\left(1/3\right)(2\cos\sqrt{3t}+1)$.  Table 5 gives a comparison between the numerical results of *y*(*t*) in some points of the interval [0,1] obtained by the method given in [8] for *N* = 2 and *M*_3_ = 11 and by the present method for *N* = 2 and *M* = 4.

## 6. Conclusion

In the present work, the hybrid of block-pulse functions with Bernoulli polynomials is used to solve nonlinear mixed Volterra-Fredholm integral equations. The problem has been reduced to a problem of solving a system of algebraic equations. The matrices *P* and *D* in (17) and (12) have large numbers of zero elements and are sparse matrices, hence, the present method is very attractive and reduces the computer memory. Illustrative examples are given to demonstrate the validity and applicability of the proposed method.

## Conflict of Interests

The authors declare that there is no conflict of interests regarding the publication of this paper.

## Figures and Tables

**Table 1:** 

*t*	Method in [14] with *N* = 2 and *M*_1_ = 4	Present method with *N* = 1 and *M* = 4
0.0	3.1021*e* − 5	1.3322*e* − 15
0.2	3.2341*e* − 6	1.3322*e* − 15
0.4	1.9092*e* − 5	1.1102*e* − 15
0.6	1.5029*e* − 5	9.9920*e* − 16
0.8	3.6499*e* − 6	7.7715*e* − 16
1.0	2.4290*e* − 5	2.2204*e* − 16

**Table 2:** 

Methods	||*y*−*y*_*e*_||_*L*^∞^(0,1)_
Method of [14]	
*N* = 2, *M*_1_ = 5,	<10^−7^
*N* = 3, *M*_1_ = 5,	<10^−8^
*N* = 4, *M*_1_ = 5,	<10^−9^
Present method	
*N* = 2, *M* = 5,	<10^−9^
*N* = 3, *M* = 5,	<10^−11^
*N* = 4, *M* = 5,	<10^−11^

**Table 3:** 

*t*	Adomian method	Method in [14] with *N* = 4 and *M*_1_ = 7	Present method with *N* = 4 and *M* = 4
0.0	0.006048	0.0060483739	0.0060483739
0.2	0.018192	0.0181929364	0.0181929364
0.4	0.030424	0.0304246702	0.0304246702
0.6	0.042669	0.0426691183	0.0426691183
0.8	0.054371	0.0543716533	0.0543716533
1.0	0.061458	0.0614587374	0.0614587374

**Table 4:** 

*t*	Method in [8] with *N* = 2 and *M*_3_ = 11	Present method with *N* = 2 and *M* = 4	Exact solution
0.0	1.00000000000	1.00000000000	1.00000000000
0.0625	1.13314845282	1.13314845305	1.13314845306
0.1250	1.28402541665	1.28402541667	1.28402541668
0.1875	1.45499141433	1.45499141461	1.45499141461
0.2500	1.64872127062	1.64872127078	1.64872127070
0.3125	1.86824595710	1.86824595741	1.86824595743
0.3750	2.11700001648	2.11700001668	2.11700001661
0.4375	2.39887529357	2.39887529393	2.39887529396
0.5000	2.71828182826	2.71828182842	2.71828182845
0.5625	3.08021684845	3.08021684898	3.08021684891
0.6250	3.49034295717	3.49034295742	3.49034295746
0.6875	3.95507672235	3.95507672297	3.95507672292
0.7500	4.48168906993	4.48168907038	4.48168907033
0.8125	5.07841903648	5.07841903712	5.07841903718
0.8750	5.75460267546	5.75460267603	5.75460267600
0.9375	6.52081911946	6.52081912035	6.52081912033
1.0000	7.38905609819	7.38905609894	7.38905609893

**Table 5:** 

*t*	Method in [8] with *N* = 2 and *M*_3_ = 11	Present method with *N* = 2 and *M* = 4	Exact solution
0.0	1.0000000000	1.0000000000	1.0000000000
0.0625	0.9990222526	0.9960975654	0.9960975632
0.1250	0.9844055237	0.9844359392	0.9844359398
0.1875	0.9680159682	0.9651516526	0.9651516562
0.2500	0.9383506092	0.9384704781	0.9384704793
0.3125	0.9074518600	0.9047047767	0.9047047741
0.3750	0.8639866978	0.8642498478	0.8642498461
0.4375	0.8201591812	0.8175793124	0.8175793136
0.5000	0.7647877177	0.7652395632	0.7652395632
0.5625	0.7102158051	0.7078433572	0.7078433525
0.6250	0.6453877501	0.6460626343	0.6460626367
0.6875	0.5827577851	0.5806206938	0.5806207020
0.7500	0.5113646167	0.5122836956	0.5122836972
0.8125	0.4437393774	0.4418516716	0.4418516650
0.8750	0.3689792921	0.3701491788	0.3701491750
0.9375	6.2996549401	0.2980156676	0.2980156705
1.0000	0.2248834557	0.2262956409	0.2262956409
